# Editorial: Special Issue “Biomarkers in Interstitial Cystitis/Bladder Pain Syndrome (IC/BPS)”

**DOI:** 10.3390/diagnostics12071689

**Published:** 2022-07-11

**Authors:** Jochen Neuhaus, Andreas Gonsior, Mandy Berndt-Paetz

**Affiliations:** 1 Research Laboratory, Department of Urology, University of Leipzig, 04103 Leipzig, Germany; mandy.berndt@medizin.uni-leipzig.de; 2 Department of Urology, University Hospital Leipzig AöR, 04103 Leipzig, Germany; andreas.gonsior@medizin.uni-leipzig.de

Interstitial Cystitis/Bladder Pain Syndrome (IC/BPS) is a disabling chronic disease of still unknown origin and complex pathophysiology. The disease affects mainly female patients, with a female to male ratio of about 9 to 1. Prevalence ranges from 52 to 500/100,000 in females and 8 to 41/100,000 in males. The diagnosis of IC/BPS is mainly hampered by the lack of appropriate biomarkers and, therefore, extensive clinical examinations are required to exclude “confusable” diseases [1]. In consequence, most patients experience several years of ineffective treatments of various urinary tract symptoms often associated with, but by themselves not characteristic of, IC/BPS. Unequivocal diagnosis of IC/BPS is the prerequisite to find more effective therapeutic approaches. Therefore, more specific biomarkers are needed to facilitate IC/BPS diagnosis and to stratify patients for treatment at earlier stages of the disease. In this Special Issue, we gathered reviews and original work elucidating the current developments in IC/BPS biomarker research.

Abreu-Mendes and coworkers explored in their systematic review if urinary excretion of vascular endothelial growth factor (VEGF), which can enhance bladder pain, could serve as biomarker of IC/BPS. They found that there is good evidence that VEGF is related to IC/BPS pain symptoms. However, the literature data are still heterogenic, and the number of patients over all included is small, but VEGF and its receptors should be included in future biomarker studies to generate a larger database [2].

Although the clinical hallmarks of IC/BPS are persisting pelvic pain in combination with unspecific lower urinary tract symptoms, such as urinary frequency and urgency, cystoscopically evident pathological alterations characterize the classical form of IC/BPS described by Guy LeRoy Hunner as early as 1915 [3]. There is growing evidence for the view that Hunner-type IC/BPS (HL) is a distinct form of chronic bladder inflammation, characterized by enhanced local immune response and urothelial denudation. 

In his review, Yoshiyuki Akiyama explored if distinct biomarkers are needed for the Hunner-type IC/BPS and the non-Hunner-type IC/BPS (NHL) [4]. He presents support for the view that these are different diseases of the bladder, HL with bladder etiology, representing a chronic inflammation of the bladder, and NHL residing within the so-called functional somatic syndromes with relations to the bladder but non-inflammatory. In consequence, biomarkers of IC/BPS can be classified in respect to either unspecific detecting HL and NHL or more specific for either subtype of IC/BPS (Table 1, [4]). Most interesting, Akiyama identified only two biomarkers specific of NHL, elevated levels of the nerve growth factor (NGF), and platelet-derived endothelial cell growth factor (PD-ECGF), highlighting a possible neural involvement (NGF) and the disturbance of angiogenesis (PD-ECGF). In summary, more specific biomarkers are emerging for HL than for NHL and most biomarkers are not validated reliable.

Lee and coworkers add an interesting new aspect to this problem as they investigated ß-defensin2, which is an antimicrobial peptide normally expressed in the bladder upon inflammation and persistently expressed in IC/BPS [5]. In their study, the authors compared the urine BD-2 levels in three female groups, normal controls, non-Hunner-type IC (NHIC) and Hunner-type IC (HIC). They found significant higher BD-2 levels in the HIC group than in the control or NHIC. Those expression levels correlated with higher mast cell counts in HIC. These finding further support our understanding of HIC as a chronic inflammation condition, possibly altering the bladder microbiota.

Bschleipfer and Karl further elucidate the possible role of the microbiome in IC/BPS, asking if there is a potential for the development of new biomarkers from microbiome in ulcerative, Hunner-type IC [6]. The authors emphasize that state-of-the-art sequencing and culture techniques have shown that the bladder is not a sterile niche, but the bladder/urinary microbiome reflects healthy and pathological body conditions. However, data on specific alterations associated with IC/BPS are still conflicting and the few data on HIC vs. NHIC patients do not provide evidence for a microbiome-based etiology of HIC. In addition to the high diversity of the microbiome, methodical problems may cause contradicting data. The authors conclude that at present the urinary microbiome cannot provide valid biomarkers for diagnostic or prognostic purposes of IC/BPS.

Questioning if urinary biomarkers can discriminate between Hunner-type IC (HIC) and non-Hunner-type (NHIC) patients, Jiang et al., analyzed the urinary cytokine/chemokine profiles of a large cohort of consecutive patients with clinically diagnosed IC/BPS (*n* = 309) and healthy controls (*n* = 30) [7]. The urine levels of five proteins were different between IC/BPS and controls, but only MIP-1β and TNF-⍺ had an AUC > 0.7 in separation of IC/BPS from healthy controls. Another five proteins separated the HIC from the NHIC group. Interestingly, two NHIC-subgroups as defined by the maximum bladder capacity (MBC, cutoff 760 mL) showed distinct levels of eight proteins. These findings support the recent view that the ulcerative subtype of IC/BPS (HIC) and the non-Hunner-type (NHIC) are different diseases, and further imply that NHIC represent several subtypes of IC/BPS. Despite the sensitivity and specificity of the urine biomarkers was not sufficient to differentiate between HIC and NHIC, urinary and serum biomarkers can provide valuable information to further diagnostic steps. Therefore, the authors conclude that future diagnosis of IC/BPS should be based on both, clinical symptoms and urinary or serum biomarkers. Furthermore, these findings could be a directive to improve the still insufficient treatment of NHIC.

Alterations of the urothelium are a hallmark of IC/BPS, ranging from cystoscopically nonsuspicious to ulcerative Hunner-type IC. Reduced proliferation and self-repair and partial urothelial denudation are well documented. The dysfunction of the urothelial barrier includes an impaired urothelial glycosaminoglycan (GAG) and glycoprotein layer. Unfortunately, our knowledge on the glycobiology and pathology of IC/BPS is still rudimentary. In their experimental study, Peskar et al. investigated the glycosylation patterns in normal and pathologic human urothelium, and experimental models of IC/BPS [8]. They found that labeling with Jacalin, a lectin with preferred binding to GalNAc was significantly diminished in the urothelium of IC/BPS patients compared to controls. Furthermore, a cyclophosphamide (CYP) induced bladder inflammation mouse model showed similar diminishing of Jacalin labeling. The authors conclude that specific changes in the glycosylation pattern may contribute to IC/BPS pathology and that the CYP-mouse model can be used to investigate this part of IC/BPS pathology.

Sultana et al. contribute a review on the endogenous cannabinoid system (ECS) in the bladder and its possible role in IC/BPS [9]. This lipid signaling system is involved in bladder neuronal sensory and pain signaling, but the cannabinoid receptors CB1R and CB2R were also detected on urothelial cells. The authors evaluated the findings on ECS modulation in experimental IC/BPS animal models. They conclude that despite there is good evidence for ECS related IC/BPS pathology, more and improved animal studies and clinical studies are needed to identify possible ECS biomarkers.

Clinical observations imply that IC/BPS develops over a long time and that the symptoms in early stages are mostly misdiagnosed as related urinary tract diseases, such as overactive bladder or chronical prostatitis. Biomarkers able to discriminate between confusable diseases and IC/BPS at early stages would be of great value for early onset of specific treatment. Our group, therefore, reviewed literature for biomarkers possibly shedding some light on the etiopathology of IC/BPS [10]. We found good evidence for HIC and NHIC being distinct entities of IC/BPS with different etiopathologies. Genetic alterations relate IC/BPS to allergic diseases and this genetic predisposition may lead to unusual activation of humoral signaling cascades promoting increased leucocyte patrolling of the bladder. Recurrent urinary tract infections may also promote IC/BPS development and recent data suggest that persistent occult virus infections may contribute. Novel IC/BPS biomarkers include miRNA and lncRNA, and with further availability of the analytical technique those regulatory molecules might gain even more impact. We identified certain biomarkers of value for indicating a predisposition of IC/BPS, for early detection, differential diagnosis, and prognosis.

Although molecular biomarkers, especially in liquid biopsies, are prevalent in biomarker research, new developments in magnetic resonance imaging (MRI) technology might offer new options in non-invasive diagnosis of IC/BPS. Towner et al. evaluated the current value of MRI and the latest methodical developments in evaluation of bladder function and report on a current study of their own group on the use of contrast-enhanced (CE-) MRI to access the bladder wall hyperpermeability as one important pathological parameter in IC/BPS. They conclude that MRI-based tests would be a good tool in routine diagnosis of IC/BPS and could provide valuable information for the development of new therapeutics [11].

Finally, the overall goal is to improve the treatment of IC/BPS and as this Special Issue impressively shows, biomarkers are an important tool on this avenue. In their excellent review Lin et al. give a comprehensive overview on the currently used biomarkers in IC/BPS and evaluate their impact on the therapeutic outcome [12]. They summarize the role of biomarkers in the assessment of the treatment effects in established and experimental IC/BPS therapies, demonstrating the complexity of the syndrome, involving urothelium, neuronal afferent and efferent control, and the immune system. 

Despite our steadily increasing knowledge on the molecular and cellular mechanisms involved in the pathophysiology of IC/BPS, we are still far from understanding this disease. The great spectrum of IC/BPS biomarkers currently under evaluation raises hope that we can develop panels of biomarkers for early detection, stratification, and treatment assessment in near future. However, since IC/BPS, at least in its full-blown state, can be considered a rare disease, concerted efforts are necessary to be able to evaluate statistically robust clinical or diagnostic studies.
